# Bayesian Network Meta-Analysis of the Effectiveness of Various Interventions for Nontraumatic Osteonecrosis of the Femoral Head

**DOI:** 10.1155/2018/2790163

**Published:** 2018-08-06

**Authors:** Ji Wang, Jing Wang, Kai Zhang, Yanfang Wang, Xuanwen Bao

**Affiliations:** ^1^Department of Orthopedics, No. 210 Hospital of the PLA, Dalian, Liaoning Province 116000, China; ^2^Technical University Munich (TUM), 80333 Munich, Germany; ^3^Department of Cardiology, Sir Run Run Shaw Hospital, Zhejiang University School of Medicine, Hangzhou 310016, China; ^4^Ludwig-Maximilians-Universität München (LMU), 80539 Munich, Germany

## Abstract

**Objective:**

To assess the effectiveness of various therapeutic hip preservation strategies on patients with nontraumatic osteonecrosis of the femoral head (ONFH).

**Design:**

This is a systematic review of previous literature and in-depth Bayesian network meta-analysis of randomized controlled trials (RCTs) to compare the clinical effect of various operation methods and one physical intervention (extracorporeal shockwave).

**Data Sources:**

Electronic literature, for studies published up to December 2017, was collected from PubMed, Medline, and the Cochrane Library.

**Study Selection:**

We selected RCTs on patients with ONFH. Treatment methods included extracorporeal shockwave (ESW), core decompression (CD), multiple drilling decompression (DD), vascularized fibular grafting (VFG), free-vascularized fibular grafting (FVFG), inverted femoral head grafting (IFHG), vascular iliac pedicle bone grafting (VIPBG), osteotomy, and tantalum implantation (TI).

**Outcome:**

The primary outcome was Harris score; the secondary outcome was Harris hip score (HHS), including total hip arthroplasty requirement (THA) and progression to collapse.

**Results:**

A total of 14 randomized controlled trials were investigated. ESW had the highest improvement on Harris score (probability best 52%), followed by VFG (probability was 38%). In the meanwhile, VFG also proved to be superior in reducing the failure rates of treatment (probability lowest 59%), followed by ESW (probability lowest 24%). In femoral necrosis stage-II, VFG achieved the highest probability in preventing treatment failures (52%) and showed better performance in reducing treatment failure rates than CD.

**Conclusion:**

ESW therapy (ESWT) is the most effective intervention to improve HHS, and VFG shows superior effect on reducing treatment failure rates.

## 1. Introduction

Osteonecrosis of the femoral head (ONFH) is a debilitating disorder with a considerably high incidence in individuals aged between the third and fifth decades of life. In the United States, ONFH is reported to affect 20,000 patients each year, and it is estimated that more than 10% of ONFN patients eventually required total hip arthroplasties (THA) annually [1]. ONFH is histologically characterized by insufficient supply of blood, death of osteocytes, and bone marrow cells, as well as progressive structure damage of involved bones, which typically follows a progressive course leading to femoral head collapse and hip joint destruction. Therefore, how to retard the progression of ONFH is always a research hotspot.

The principle of ONFH treatment includes the termination of pathologic progression and the restoring of weight-bearing capacity. Nowadays, the treatment and management for ONFH consists of conservative and surgical approaches. In a large number of studies, the clinical effect of ESWT which was assessed from a comprehensive perspective showed better effect than other conservative therapeutic methods [2, 3]. Besides, some RCT researches were designed to compare different surgical interventions for ONFH, including CD, DD, bone transplantation (VFG, FVFG, VIPBG, and IFHG), Osteotomy, and TI. These surgical interventions have been applied to avoid the most invasive intervention: total hip arthroplasty (THA). Core decompression, which is strictly limited to the treatment for early stages of ONFH before femoral head collapse [4], is now one of the most frequently used interventions and offers effective outcomes. However, some studies manifested that vascularized bone grafting (e.g., VFG, VIPBG) was superior to other treatments in clinical outcomes, radiographic findings, and durability of treatments [5, 6], though the operation method of grafting process and the location of the grafts may have a significant influence on the therapeutic effect. Above all, the optimal intervention to preserve the femoral head remains controversial. In this study, a Bayesian network meta-analysis method was used for the quantitative comparison of different surgical interventions and ESWT to determine the optimal treatment for ONFH.

## 2. Method

### 2.1. Data Sources and Searches

We searched literature published between 1980 and 2017 in three electronic databases (Medline, PubMed, and the Cochrane Library) for randomized clinical trials investigating operation methods for ONFH, with Medical Subject Headings (MeSH) and text words.

For the purpose of Bayesian network meta-analysis, the search extracted trials comparing ESW, VFG, FVFG, CD, DD, IFHG, TI, osteotomy, and VIPBG. The trials involving cellular therapy or biomaterials were excluded.

### 2.2. Study Selection

We included randomized, parallel group design clinical trials comparing the effects of chosen operation methods and one physical intervention (ESW).

Inclusion criteria for studies were as follows: (1) the study should be randomized, parallel group design clinical trial; (2) the study should focus on ONFH; (3) the included studies should report at least one of the three outcomes: the improvement of Harris score and the frequency that hips required THA or underwent collapse of the femoral head after intervention.

Exclusion criteria were as follows: (1) the study was not a RCT design; (2) the study included non-ONFH patients; (3) the study combined cell-therapy effect with surgical options (e.g., VFG plus mesenchymal stem cell implantation). Eligible studies should be published as full-length articles or letters in peer-reviewed journals.

### 2.3. Data Extraction and Quality Assessment

Two investigators independently extracted the following information: study design; name of the first author, publication year; sample size, patients' characteristics; interventions; comparisons; outcomes (postoperative Harris score and treatment failure rate) and follow-up. Two investigators independently evaluated the methodological quality of eligible RCTs by the Cochrane Collaboration's tool [7] for assessing risk of bias on the basis of (1) random sequence generation, (2) allocation concealment, (3) blinding of participants and personnel, (4) blinding of outcome assessment, (5) incomplete outcome data, (6) selective reporting, and (7) other sources of bias.

### 2.4. Outcome Assessment

HHS and treatment failure rates were regarded as the outcome of the included RCTs. HHS includes four subscales: (1) range of motion, (2) joint activity, (3) pain, and (4) absence of deformity. A higher score indicates a better treatment outcome. Treatment failure rates included the frequency of THA requirement and the collapse of the femoral head after intervention. If both events were reported in RCTs, we chose the outcomes with greater number.

### 2.5. Statistical Methods

Quantitative data of each eligible study were first extracted in a spreadsheet. We analyzed two treatment outcomes separately (HHS and treatment failure rates). STATA (version 13.0, StataCorp LLC,TX, USA) and the GeMTC R package (version 3.2.3, R Development Core Team/R Foundation for Statistical Computing, Vienna, Austria) were used to perform network meta-analysis with Bayesian hierarchical random-effects model [8]. The random-effects model allowed the heterogeneity among trials on the assumption that treatment effects originated from a normal distribution. Markov Chain Monte Carlo (MCMC) simulation method was used to calculate the posterior distributions of the nodes in Bayesian network frame. Noninformative priors with vague normal (mean 0, variance 10 000) and uniform (0-2) prior distributions for outcomes such as HHS and treatment failure rates were performed [8]. Sensitivity analysis was performed to examine the effects of different operation methods. We generated 100000 simulations for each initial value, and the first 50000 simulations were discarded. Convergence was assessed by Brooks-Gelman-Rubin method. The median of the posterior was the point estimate and the corresponding 95% credible intervals using the 2.5th and 97.5th percentiles of the posterior distribution were reported as 95% credibility intervals [9]. Inconsistency was evaluated in a loop which connected three treatments [10]. We used node splitting method to evaluate the inconsistency of network model [11, 12]. A particular comparison based on direct and indirect evidence was performed, in which Bayesian P>0.05 was regarded as insignificant [12]. The probability of each treatment being the best was calculated to provide a more comprehensible measure of treatment efficacy on the basis of its posterior probability. The plots of ranking probabilities (rankograms) illustrated the order of treatments for each outcome [13].

Potential publication bias was assessed by visual inspection of comparison-adjusted funnel plot [14–16]. The Cochran's Q (X^2^) and the I^2^ statistical test were calculated to test for statistical heterogeneity [17].

## 3. Results

### 3.1. Characteristics and Quality Evaluation of Included Studies

**Figure 1:**
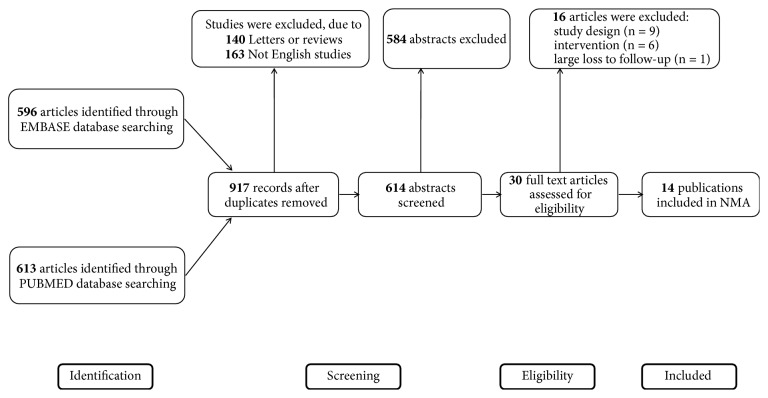
PRISMA flowchart illustrating the selection of studies included in this study.

**Figure 2:**
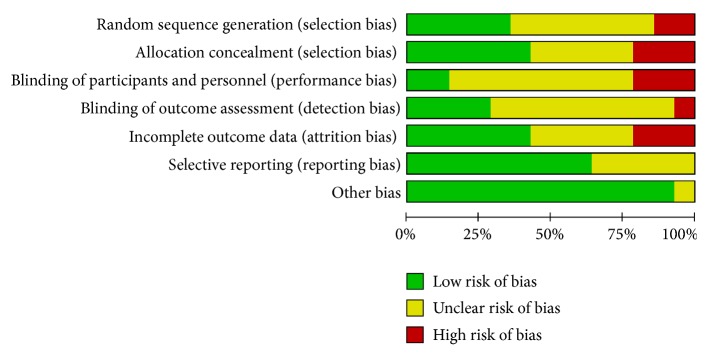
Risk of bias graph for each included study.

We retrieved 1209 related publications in total, among which 292 duplications, 140 letters or reviews, and 163 non-English publications were immediately eliminated. 584 publications were excluded by screening their titles and abstracts. The entire process of literature searching and screening for NMA was illustrated in Figure 1. With the full-text assessment, 14 RCTs with a number of 1298 patients were enrolled into our analysis. Main study endpoints included HHS, the treatment failure rates of femoral necrosis in total stage and stage-II. Baseline characteristics of the 14 studies were presented in Table 1. All of the included studies were RCTs; the overall quality of included studies was embodied in Figure 2.

### 3.2. Network Meta-Analysis of HHS

**Figure 3:**
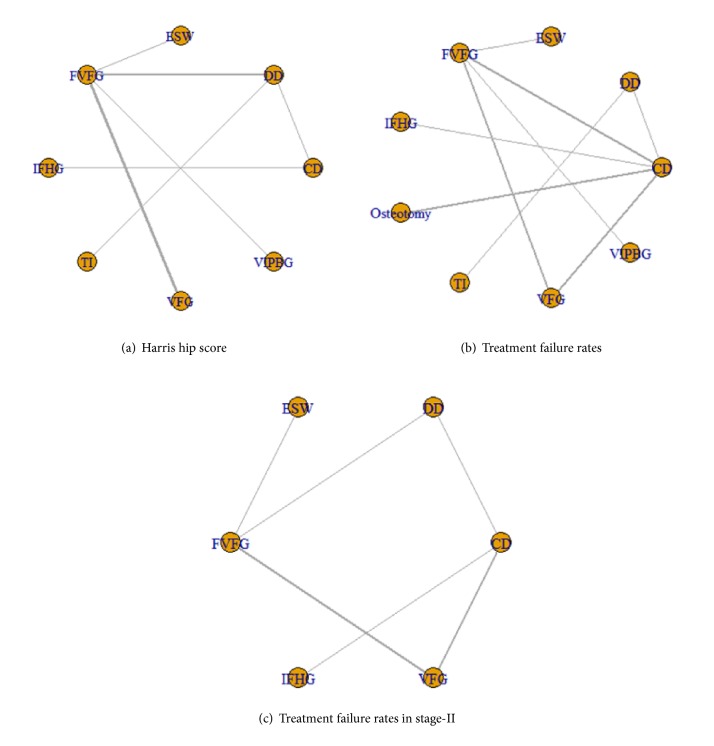
**Network comparisons for (a) HHS, (b) the treatment failure rates (Collapse & THA progression), and (c) the treatment failure rates (Collapse & THA progression) in stage-II included in the analyses**. THA: total hip arthroplasty; CD: core decompression; ESWT: extracorporeal shock wave; DD: multiple drilling decompression; VFG: vascularized fibular grafting; FVFG: free-vascularized fibular grafting; IFHG: inverted femoral head grafting (IFHG); VIPBG: vascular iliac pedicle bone grafting; TI: tantalum implantation.

10 two-arm RCT studies reported the HHS, recruiting 538 patients (Figure 3(a)). These studies were incorporated into the present network Bayesian model. The mean duration of follow-up was 22 months. VFG was the most frequently studied intervention, and the comparisons between VFG and FVFG were more than those in other studies. In the direct pairwise comparisons, enhancement for VFG was statistically significant as compared with FVFG (MD=16, 95% CrI [2.3, 29]). Other pairwise comparisons did not show significant outcome.

**Figure 4:**
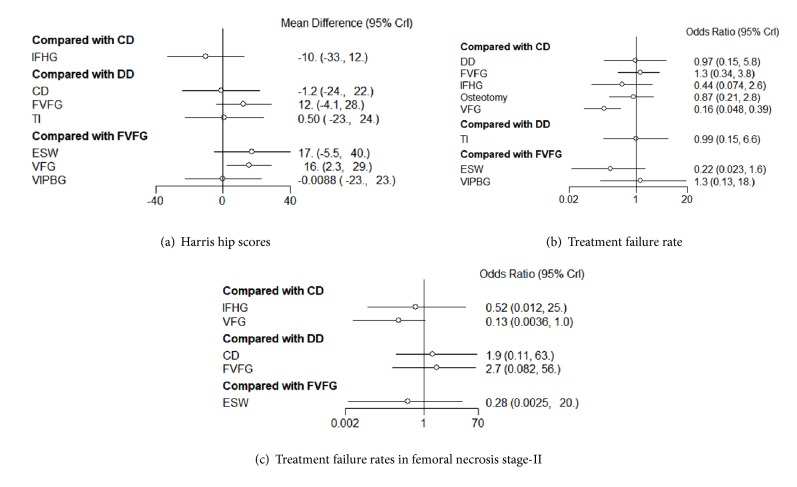
**Forest plots of direct comparison of (a) HHS and (b) the treatment failure rates included in the network meta-analysis in a Bayesian framework**. CD: core decompression; ESWT: extracorporeal shock wave; DD: multiple drilling decompression; VFG: vascularized fibular grafting; FVFG: free-vascularized fibular grafting; IFHG: inverted femoral head grafting (IFHG); VIPBG: vascular iliac pedicle bone grafting; TI: tantalum implantation.

In random-effects relative forest plots, enhancement was detected with statistical significance for ESW versus DD (MD = 29, 95% CrI [1.6, 57]), VFG versus DD, FVFG, and IFHG (MD = 28, 95% CrI [6.8, 49]; MD = 16, 95% CrI [2.3, 29]; MD = 39, 95% CrI [0.56, 78]); there was no significant effect on improving HHS among the other interventions (Figure 4(a)).

In rankograms (Figure 6(a)), ESW was estimated to have a 55.4% chance of being the best intervention for the treatments of femoral necrosis, followed by VFG (38.6%). From the perspective of improving HHS, our study revealed that ESW therapy and VFG technique were the best two options for hip preservation treatments in terms of improving HHS.

### 3.3. Network Meta-Analysis of the Treatment Failure Rates in Femoral Necrosis Total Stage

In our study, treatment failure outcomes included collapse and THA progression. There were 13 literature sources that described the treatment failure rates, recruiting 1277 patients (Figure 3(b)). In the direct pairwise comparison, the overall effect showed that VFG had a statistically lower treatment failure rate as compared to CD (OR = 0.16, 95% CrI [0.048, 0.39]), while there was no significant difference among other interventions (Figure 4(b)).

**Figure 5:**
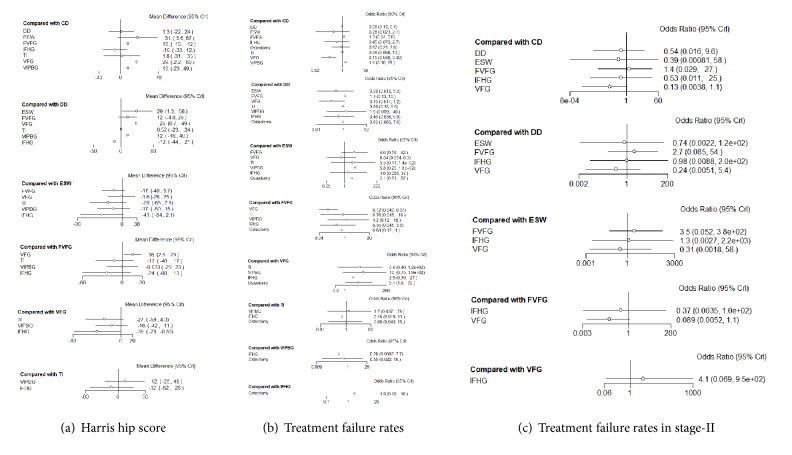
**Relative forest plots of each pairwise comparison of (a) HHS, (b) the treatment failure rates, and (c) the treatment failure rates in stage-II included in the network meta-analysis in a Bayesian framework**. THA: total hip arthroplasty; CD: core decompression; ESWT: extracorporeal shock wave; DD: multiple drilling decompression; VFG: vascularized fibular grafting; FVFG: free-vascularized fibular grafting; IFHG: inverted femoral head grafting (IFHG); VIPBG: vascular iliac pedicle bone grafting; TI: tantalum implantation.

**Figure 6:**
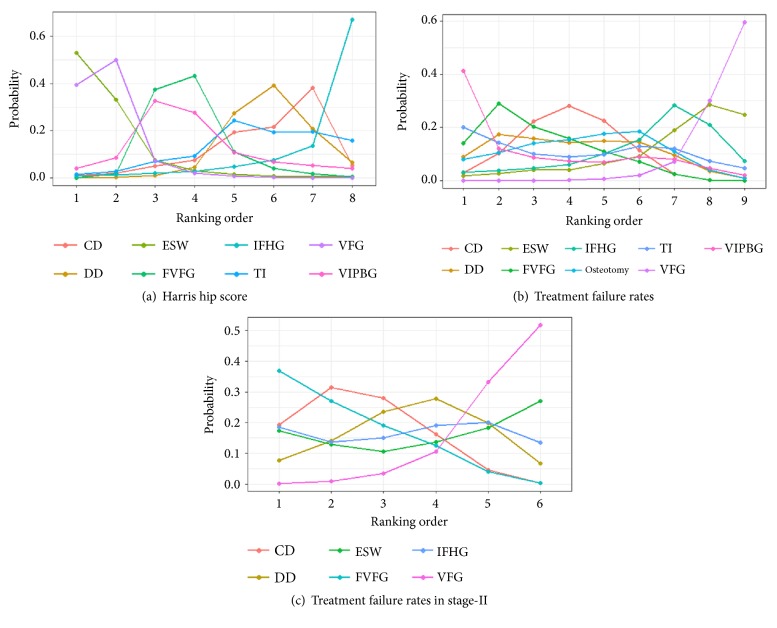
**Rank probability curves for (a) HHS, (b) the treatment failure rates, and (c) the treatment failure rates in stage-II.** Distribution of probabilities for each treatment is ranked at different positions for each outcome. CD: core decompression; ESWT: extracorporeal shock wave; DD: multiple drilling decompression; VFG: vascularized fibular grafting; FVFG: free-vascularized fibular grafting; IFHG: inverted femoral head grafting (IFHG); VIPBG: vascular iliac pedicle bone grafting; TI: tantalum implantation.

In random-effects relative forest plots, VFG had a lower treatment failure rate (OR = 0.16, 95% CrI [0.048, 0.39]; OR = 0.12, 95% CrI [0.042, 0.37]; OR = 0.18, 95% CrI [0.034, 0.94]) compared with CD, FVFG, and osteotomy for total stage (Figure 5(b)). There was no significant difference in treatment failure rates for other methods. Rankograms indicated that VFG had the lowest probability for the progression to THA and femoral collapse. Besides, ESW also showed a low probability for the progression to treatment failures (Figure 6(b)).

### 3.4. Network Meta-Analysis of the Treatment Failure Rates in Femoral Necrosis Stage-II

A total of 13 studies described the treatment failure rates in femoral necrosis stage-II (Figure 3(c)). The overall effect showed that only VFG had a statistically lower failure rate as compared with CD (OR = 0.13, 95% CrI [0.0036, 1.0]) in the direct pairwise NMA (Figure 4(c)). Moreover, we found that VFG tended to show lower failure rates than FVFG in random-effects relative forest plots (OR = 0.090, 95% CrI [0.0053, 1.1]) (Figure 5(c)). Rankograms indicated that VFG was the optimal intervention in the prevention of treatment failures (Figure 6(c)).

### 3.5. Inconsistence Check

**Figure 7:**
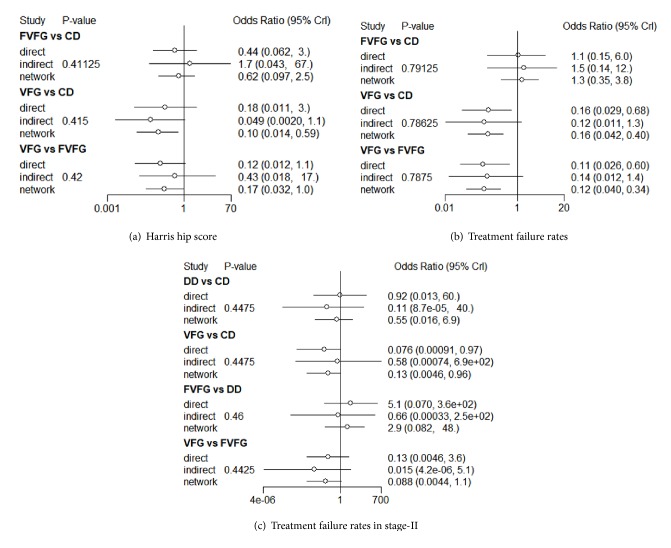
**Forest plot of network consistency analysis for comparisons by node splitting method.** Numbers represent the posterior means and standard deviations (SDs) of the direct, indirect, and network estimates of the odds ratios (ORs) and rate ratios (RRs). CD: core decompression; ESWT: extracorporeal shock wave; DD: multiple drilling decompression; VFG: vascularized fibular grafting; FVFG: free-vascularized fibular grafting; IFHG: inverted femoral head grafting (IFHG); VIPBG: vascular iliac pedicle bone grafting; TI: tantalum implantation.

Statistical consistency between direct and indirect comparisons for HHS and treatment failure rates in total stage and stage-II was analyzed in Figure 7. The overall network was highly consistent, without significant differences between direct and indirect comparisons.

### 3.6. Funnel Plot and Publication Bias

**Figure 8:**
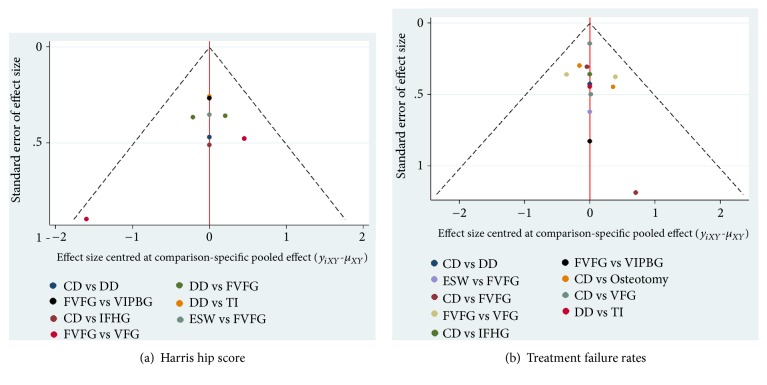
**Comparison-adjusted funnel plot for assessment of (a) HHS and (b) the treatment failure rates.** CD: core decompression; ESWT: extracorporeal shock wave; DD: multiple drilling decompression; VFG: vascularized fibular grafting; FVFG: free-vascularized fibular grafting; IFHG: inverted femoral head grafting (IFHG); VIPBG: vascular iliac pedicle bone grafting; TI: tantalum implantation.

The funnel plot of HHS and treatment failure rates were shown in Figures 8(a) and 8(b), respectively. Symmetrical Scatters were observed in the funnel plot, manifesting the publication bias in the results of HHS, and treatment failure rates events in various studies were relatively low.

## 4. Discussion

How to postpone ONFH progression remains a challenge that needs to be fully understood. Although there are varieties of treatments for preserving the femoral head [18], the optimal one is still controversial. In the present Bayesian network meta-analysis study, we systematically analyzed the effects of several hip preservation treatments on nontraumatic ONFH, including ESW, CD, DD, VFG, FVFG, IFHG, VIPBG, osteotomy, and TI.

CD is a standard technique widely used in patients with early-stage ONFH, which helps to decrease intraosseous pressure in the femoral head, reestablish vascular flow, and relieve pain [19]. There were many studies that investigated the efficacy of CD for the treatment of patients with early-stage ONFH. Nevertheless, the clinical success of CD has inconsistencies (from 47% to 83%), mainly related to many different factors including causes and the stage of the lesion, skill of procedures, and duration of follow-up [20–23]. Based on our network results and rankings results, CD provided a moderate outcome in terms of improving HHS and preventing treatment failures as compared with other joint-preserving treatments.

Some articles indicated that the conventional core decompression might lead to further collapse of femur head due to the weakening of subchondral bone support [24, 25]. To overcome the limitations of this surgery, multiple drilling has been advocated. One research demonstrated that multiple drilling effectively reduced the pressure of femoral head and meanwhile maintained the supportive role of the subchondral bone to avoid fractures or collapse of the femoral head [26]. However, DD did not show significant better effect on both the improvement of HHS and the prevention of THA progression than CD in our network study, though DD had a higher probability to rank better from rankograms in these two aspects.

Based on our network results and rankograms, we recommended ESW as one of the optimal interventions due to its better efficacy and moderate safety in improving HHS. The mechanism of ESW in the treatment of ONFH has not been fully understood [2]. ESW was equipped to spread to necrotic femoral hips, and a pressure loss of 50% of shockwave was observed [27, 28]. Some researches proposed that ESW could induce microfracture to accelerate bone healing and enhance pain threshold [29–31]. Furthermore, ESW promoted bone healing by enhancing osteogenesis and angiogenesis as well as bone remodeling of diseased hips [32, 33]. In this study, rankogram of ESW showed the highest probability (52%) of being the best treatment in terms of HHS improvement. Increased range of motion and joint activity as well as reduction of pain might be due to the promotion of tissue remolding and the bluntness of pain sensation, which would help the improvement of HHS. For the prevention of collapse and THA progression, ESW also had a satisfactory performance both in all stage and stage-II in rankogram, indicating that ESW was one of the most optimal ways in inhibiting progression.

The use of vascularized fibular graft was initiated in an effort to arrest the progression of necrosis and to enhance angiogenesis. The success of this surgical method was in connection with multiple factors: (1) sufficient decompression, (2) mechanical support, and (3) augmentation with additional cancellous bone graft. VFG not only provides structural support, but also restores vascular supply to enhance lesion healing [34, 35]. A report on the long-term results of VFG indicated that 49 of 65 hips (75%) survived at a mean follow-up of fifteen years (range: 10.5–26.1 years) [36]. Moreover, some studies suggested that VFG was a better treatment option than CD and nonvascularized fibular grafting due to the less dome depression of the femoral head [37–39]. Our results showed VFG had significantly better effect in improving HHS than DD and FVFG. Besides, it also had a better outcome in terms of inhibiting collapse and THA progression than DD, FVFG, and osteotomy. Meanwhile, the rankogram showed VFG had the highest probability (59%) of being the best treatment in preventing collapse and THA progression. In HHS improvement, VFG had 39% probability of being the best treatment and 50% probability of being the second best treatment. Hence, we got the conclusion that VFG would be one of the most optimal interventions.

TI has been used as reasonable mechanical substitute of a fibular graft following CD for ONFH treatment [40]. The tantalum implant aims to provide a structural scaffold for bone ingrowth by its osteoconductive capacity [41]. However, the effect of TI transplantation is still under debate. In one study, 16 of 58 hips (28%) showed radiographic progression and 9 of 58 hips (16%) converted to THA at a mean follow-up of 24 months [42]. Tanzer M found the presence of bone ingrowth in thirteen (87%) of the fifteen specimens by backscattered scanning electron microscopy. The mean extent of bone ingrowth was only 1.9% (range: 0% to 4.4%). The retrieved implants were associated with limited bone ingrowth and insufficient mechanical support of subchondral bone [43]. Based on our study, TI did not have significantly different effect as compared with DD and CD, yet the effect of TI, DD, and CD was not as good as that of ESW and VFG.

Osteotomies aim to prevent femoral head collapse by transposing the osteonecrotic area from a weight-bearing to a non-weight-bearing area of the hip joint, thereby diverting mechanical stress from the lesion to healthy bone. An earlier study found that 28 hips (76 %) treated by intertrochanteric varus osteotomy had a good or excellent result at a mean follow-up of 11.5 years [44]. Another study indicated that 22 of 28 hips (79 %) treated by intertrochanteric rotational osteotomy had survived with a mean HHS of 85.8 at the final follow-up [45]. In recent years, the usage of osteotomy has been reduced due to limited indications for small lesions. Besides, osteotomies were associated with a higher rate of complications, such as nonunion or delayed union and loss of fixation and/or position, which also restricted the usage of this technique [46]. Moreover, this surgical technique was highly complicated, and it was difficult to convert the failed cases to THA. Based on the available data from our network analysis, we do not recommend this technology as the first choice until the publication of long-term follow-up results.

IFHG and VIG are two alternative interventions for ONFH. When IFHG was used, one cylindrical block of cancellous bone was harvested from the femoral head and then inverted and reinserted into the core. One study showed the improvement of HHS by IFHG compared with CD, although a proportion of patients experienced radiographic progression [47]. When VIPBG technique was used, vascularized iliac bone grafting was taken to replace the necrosis region [48]. In our study, the two interventions did not show significant difference in preventing collapse compared with other interventions. In terms of HHS, VFG showed slightly better outcome than IFHG. Thus, we would recommend VFG as a preferred choice for ONFH treatment compared with IFHG and VIG.

As far as we know, this was the first comprehensive network comparison of mainstream interventions for ONFH. To compare the efficacy of nine treatments for ONFH, we used a Bayesian network model to combine direct and indirect evidence on the effectiveness in 14 RCTs. From the analysis, we got the conclusion that VFG and ESW would be the optimal choices for the treatment of early ONFH in terms of improving HHS and preventing treatment failures. We also analyzed the patients by disease stage to get a better understanding of the efficacy of various interventions at different disease stages. As far as preventing treatment failures was concerned, VGF and ESW therapy also showed better outcomes in stage-II.

Nevertheless, this study had several limitations. First of all, only published studies were included in the present meta-analysis. Thus, publication bias may have occurred. Secondly, the heterogeneity existed in terms of risk factors, lesion sizes and stages, bilateral versus unilateral disease, surgical techniques, indications for THR, and follow-up time. Thirdly, detailed blind methods and allocation concealment were not described in some of the included RCTs, which could affect the validity for overall findings. Furthermore, due to the different classification measurements (radiographic evidence based on X-ray and MRI) applied in various reports, the reliability of the avascular necrosis status assessment was decreased.

## Figures and Tables

**Table 1:** Design and characteristics of randomized controlled trials (RCTs) included in the Bayesian network meta-analysis.

N	Author	Year	NCT register	Comparisons (Group A vs Group B)	No. of patients	No. of Hips	Mean age	Stage of patients	Follow-up, months
1	Asser A. Sallam	2017	NA	CD vs IFHG	61	A:38 B:33	A:33.21 ± 8.79 B:32.76 ± 8.16	Ficat I -III	94.32(36-168)
2	Cao	2017	NA	DD vs FVFG	21	42	31±6(21–48)	ARCO I - III	6,12,24,36
3	S. P. Mohanty	2016	NA	DD vs FVFG	46	69	35.38±7.55	Ficat I – III	3,6,12,24
4	Miao	2015	NA	DD vs TI	A:30 B:30	A:34 B:36	A:35.2±5.8 B:32.6±6.3	Steinburg I -II	A:18.1±5.2 B:19.8±4.1
5	Abdullah Al Omran	2013	NA	CD vs DD	A:61 B:33	A:61 B:33	26 (15-33)	Ficat I -I II	3,6,12,24
6	Cihangir TETIK	2011	NA	VFG vs FVFG	A:8 B:13	A:11 B:15	A:34(30-40) B:34 (17–66)	Ficat II-IV	A:22(12-57) B:42(16–114)
7	Cheng-Yo Yen	2006	NA	FVFG vs VIPBG	A:22 B:33	A:22 B:39	A: 38(28-52) B: 40(26–63)	Steinburg I-IV	A:36 B:48
8	Wang	2005	NA	ESW vs FVFG	A:23 B:25	A:29 B:28	A:39.8 ± 12.1(19-63) B:39.9 ± 9.3(19-53)	ARCO I-III	A: 25.2 ± 3.7 (24-38) B: 25.8 ± 4.6 (24-39)
9	SHIN-YOON KIM	2005	NA	VFG vs FVFG	A:19 B:23	A:19 B:23	A:43(24-52) B:44(23-51)	Steinburg II-IV	A:50(36-66) B:50(36-67)
10	ANTON Y.PLAKSEYCHUK	2003	NA	VFG vs FVFG	A:50 B:50	A:50 B:50	A:44(23-52) B:44(16-67)	Pittsburgh I-III	A:60 B:60
11	Hans-Georg Simank	2001	NA	Osteotomy vs CD	A:67 B:74	A:83 B:94	A:42(21-60) B:40(16-77)	Steinburg I -V	A:72 B:72
12	Scully SP	1998	NA	VFG vs CD	A:480 B:72	A:614 B:98	A:35(18-60) B:41(18-66)	Ficat I-III	A:50 B:50
13	KYUNG-HOI KOO	1995	NA	CD vs VFG	33	A:19 B:18	47(18-68)	Steinburg I-III	24
14	Steve M	1993	NA	CD vs FVFG	A:15 B:19	A:19 B:20	42(26-48)	Ficat II-III	24(24-60)
